# Dissection of Drought Tolerance in Upland Cotton Through Morpho-Physiological and Biochemical Traits at Seedling Stage

**DOI:** 10.3389/fpls.2021.627107

**Published:** 2021-03-12

**Authors:** Zobia Zahid, Muhammad Kashif Riaz Khan, Amjad Hameed, Muhammad Akhtar, Allah Ditta, Hafiz Mumtaz Hassan, Ghulam Farid

**Affiliations:** ^1^Plant Breeding and Genetics Division, Nuclear Institute for Agriculture and Biology, Faisalabad, Pakistan; ^2^NIAB-C, Pakistan Institute of Engineering and Applied Science Nilore, Islamabad, Pakistan; ^3^Soil and Environmental Sciences Division, Nuclear Institute for Agriculture and Biology, Faisalabad, Pakistan

**Keywords:** *Gossypium*, water stress, stress tolerance indices, root length, stomatal conductance, catalase

## Abstract

Cotton is an important fiber and cash crop. Extreme water scarceness affects the growth, quality, and productivity of cotton. Water shortage has threatened the future scenario for cotton growers, so it is imperative to devise a solution to this problem. In this research, we have tried to machinate a solution for it. 23 genotypes have been screened out against drought tolerance at the seedling stage by evaluating the morphological, physiological, and biochemical traits in a triplicate completely randomized design plot experiment with two water regimes [50 and 100% field capacity]. Genotypic differences for all the morphological and physiological traits revealed highly significant differences except transpiration rate (TR). Moreover, the interaction between genotype and water regime (G × W) was highly significant for root length (RL, 5.163), shoot length (SL, 11.751), excised leaf water loss (ELWL, 0.041), and stomatal conductance (SC, 7.406). A positively strong correlation was found in TR with relative water content (RWC; 0.510) and SC (0.584) and RWC with photosynthesis (0.452) under drought conditions. A negative correlation was found in SC with SL (−0.428) and photosynthesis (−0.446). Traits like RL, SL, SC, photosynthesis, proline, catalase, and malondialdehyde were visible indicators, which can differentiate drought-tolerant genotypes from the susceptible ones. A wide range of diversity was found in all the morpho-physiological traits with the cumulative variance of four principal components (PCs) 83.09% and three PCs 73.41% under normal and water-stressed conditions, respectively, as per the principal component analysis. Hence, selection criteria can be established on the aforementioned traits for the development of drought-tolerant cultivars. Moreover, it was found that out of 23 experimental varieties, NIAB-135, NIAB-512, and CIM-554 could be used to devise breeding strategies for improving drought tolerance in cotton.

## Introduction

*Gossypium* species exhibit prodigious morphological variation, ranging from trailing herbaceous perennials to ∼15-m trees with a diverse array of reproductive and vegetative characteristics. Mostly, commercially cultivated cotton varieties are derived from two species, *G. hirsutum*, and *G. barbadense. G. hirsutum* is the most cultivated species as it contributes to 90% of the world’s total cotton production (Hu et al., 2019).

A decrease in cotton production was observed from 13.960 million bales to 11.935 million bales in the last 5 years, observing a decline of 14% (Anonymous, 2017). Drought greatly affects crop productivity, root proliferation and its systems, and plant diseases and surges insect invasion. A 34% decrease in cotton production in Pakistan was observed from the previous year because of heat and water stress (Ullah et al., 2017). The effect of drought on cotton, like other crops, is widespread and varies from environment to environment, but it is affecting productivity enormously. Drought stress affects the physiology of plants by cellular and molecular mechanisms (Chaves et al., 2003). The demand for drought-tolerant genotypes will be intensified as water resources are becoming scarce with time. Being a glycophyte, cotton exhibits higher tolerance toward abiotic stresses as compared to other major crops. However, extreme environmental conditions, such as drought, affect the growth, productivity, and fiber quality of cotton (Parida et al., 2008).

Drought tolerance mechanisms in plants are classified into four categories: drought avoidance, drought tolerance, drought recovery, and drought escape (Fang and Xiong, 2015). Two main approaches of plants against drought stress are drought avoidance and drought tolerance. The erraticism in the cotton crop is limited as far as drought tolerance is concerned. The tolerance variability is available at the seedling stage. It is very important to know how plant responds and behaves in order to work on the development of drought-tolerant genotypes. Different morphological traits could be used to classify drought sensitivity and tolerance in upland cotton (Jaleel et al., 2009). Significant differences had been reported in various morphological traits such as shoot length (SL), number of bolls per plant, plant height, root length (RL), and boll weight (Mahmood et al., 2006). Root morphology plays a vital role in drought response determination (Başal and Ünay, 2006; Liu et al., 2008). Leaf water content is another factor for the determination of drought tolerance in plants. Usually, plants with high water content are drought-tolerant plants (de Brito et al., 2011). The stress susceptibility index (SSI) was introduced by Maurer and Fischer, which is now used for screening of genotypes against different stresses (Fischer and Maurer, 1978). SSI lesser than a unit value indicates that a variety is drought tolerant, and if the value is more than a unit, it indicates that a variety is drought sensitive (Guttieri et al., 2001). Fernandez introduced the stress tolerance index (STI), which is very useful in recognizing varieties with high potential of yield under drought and controlled conditions (Fernandez, 1992).

The transpiration rate (TR) and stomatal conductance (SC) are stimulated by hormonal and chemical signaling, and studies have revealed that under drought conditions, the SC and transpiration decrease, which could be used for screening of germplasm against drought tolerance (Sabatini et al., 1999). Since the pool of germplasm exhibits variable response under control or stressed conditions, there is a dire need for regular screening for better adaptability and viable production under drought stress. Photosynthesis was controlled by both non-stomatal and stomatal factors. The photosynthetic rate was normally reduced under drought stress. The drought-tolerant species did some carbon fixation by regulating stomatal movements in order to improve the water use efficiency or open stomata only when water deficiency is over (Lawlor and Cornic, 2002). Photosynthesis reduced under drought stress as a result of damage in photosynthetic machinery, leading to leaf aging at an early age, decline in leaf expansion and surface area, and decrease in food production (Wahid and Rasul, 2005). Drought also forces the plant to close the stomata, which causes a reduction in CO_2_ uptake and makes the plant susceptible to photodamage (Cornic and Massacci, 1996).

At the seedling stage of cotton, relative water content (RWC) is a selection criterion for drought-tolerant genotypes. Cotton is more sensitive to low water potential for photosynthesis in comparison to SC (Krieg, 1986), which is due to a reduction in the synthesis and activity of photosynthetic enzymes under drought conditions (Jones, 1973). Drought stress decreases cell membrane stability, chlorophyll a and b, dry matter stocking, and RWC in cotton (Gadallah, 1995). Studies of cotton genotypes at different water stress levels exhibited that the water content of leaf and quantum yield of photosystem-II decrease with an increase in drought stress (Wang et al., 2007). Water shortage disturbs cellular growth (Turner et al., 1986; Wang et al., 2007), hinders leaf and stem elongation (Jordan, 1970), and decreases the number of floral buds (McMichael and Hesketh, 1982; Ball et al., 1994; Gerik et al., 1996). Cell expansion is directly influenced by leaf water content (Schonfeld et al., 1988). As a result of a reduction in cell expansion, the growing rate of stems and roots also reduces, and this eventually reduces cotton yield (Hale and Orcutt, 1987; Ball et al., 1994; Pace et al., 1999; Pettigrew, 2004; Babar et al., 2009). Due to drought, reactive oxygen species (ROS) and antioxidant production balance are disturbed, which may cause ROS accumulation in plant systems (Fu and Huang, 2001; Reddy et al., 2004).

Keeping in view the importance of drought effects in cotton, this study was designed to identify the indices and genetic sources of drought tolerance in cotton at the seedling stage, which is the most critical stage for proper plant development and its systems. The identified genotypes along with stress tolerance indices will help to design breeding strategies to improve drought tolerance in cotton to meet the future demand of climate-smart crops.

## Materials and Methods

To conduct the present study, healthy seeds of 23 genotypes were collected from Ayub AgricuItural Research Institute (AARI), Cotton Research Institute (CRI), Central Cotton Research Institute (CCRI), Multan Nuclear Institute for AgricuIture and Biology (NIAB), and University of AgricuIture Faisalabad (UAF; Table 1).

**TABLE 1:** List of genotypes used in the experiment.

Sr. No.	Genotypes	Sr. No.	Genotypes
1.	FH-326	13.	SLH-375
2.	FH-490	14.	VH-363
3.	FH-498	15.	MARVI
4.	CIM-496	16.	Gomal-105
5.	CIM-343	17.	MNH-786
6.	CIM-554	18.	NIBGE-2
7.	CIM-663	19.	NIAB-112
8.	BH-160	20.	NIAB-135
9.	BH-167	21.	NIAB-512
10.	BH-199	22.	NIAB-852
11.	FH-142	23.	NIAB-846
12.	FH-Lalazar		

The trial was conducted in triplicate completely randomized design (CRD). First of all, soil analysis was carried out for the field capacity (FC) determination, elemental analysis of soil, electrical conductivity, pH, organic matter, and soil texture. The values of the characteristics determined are mentioned in Table 2.

**TABLE 2:** Analysis of soil being used in the experiment.

Soil characteristics	Values	Soil characteristics	Values
Saturation percentage	40.9	CO_3_	NIL
pH	7.8	HCO_3_^−^ (meq L^–1^)	2.5
EC (dSm^–1^)	1.5	Soil texture	Silt loam
Organic matter (%)	0.74	Ca^2 +^ + Mg^2 +^ (meq L^–1^)	25
Na^+^ (mg Kg^–1^)	67	Available K (mg Kg^–1^)	16
Chlorite (meq L^–1^)	1.5	Ca^2 +^ (meq L^–^)	20
Field capacity (ml)	408		

Seeds were planted in November 2018 in polythene bags (30 × 14 cm). The bags were filled with about 1.6 kg of silt and watered to FC before sowing. Seeds were soaked overnight. The next morning, three–four seeds were sown approximately 2 cm deep in each polythene bag. After germination, thinning was done to maintain one seedling per bag. Glasshouse temperature was kept at ∼35 and ∼28°C using cooling and heating systems. Electric bulbs were used to maintain daylight intensity at 2,500 lux for 14 h. Nitrogen at the rate of 0.2 *g* urea was supplied after 14 days of sowing, and seedlings were watered for their good development. Two moisture levels were kept. The weight of soil moisture at FC was calculated as the difference between the soil weight after drainage and soil weight after oven drying for 105°C for 24 h. Eighteen polythene bags of each cultivar were divided into two sets, i.e., normal (N) and drought (D) treatments accounting for 03 bags per replicate. The two sets were watered at 100% FC daily until the development of second true leaf. Drought (D) was imposed at this stage. The normal (N) set was watered to maintain a 100% FC when the water content in soil reached the maximum allowable deficit (MAD; that is, 50% of the FC). However, the seedlings kept under drought stress were maintained at 50% FC for 10 days. Polythene bags were weighed daily and seedlings were watered accordingly. The experiment continued until the fourth main stem leaf, and at this stage, young plants were uprooted for the measurement of morpho-physiological parameters (Hassan et al., 2015).

### Morphological Parameters

When the fourth true leaf appeared, data on morphological parameters, i.e., RL, SL, RWC, and excised water loss, were recorded for analysis. Shoots of each genotype were taken and rinsed with distilled water thoroughly. Then, the shoot parts were detached and the length was measured in centimeters. The mean SL for each genotype was calculated in each treatment for analysis. Roots of each genotype were also taken, washed with distilled water, and measured in centimeters. The mean RL for each genotype was computed.

### Physiological Parameters

Physiological parameters including photosynthesis, TR, and SC were measured by using LI-1600 Steady-State Porometer. Data were recorded for both control and drought groups on sunny days between 11:00 am and 2:00 pm. The readings were taken from the third leaf of every replicate of a particular genotype (Singh et al., 2018). RWC and leaf water loss are the physiological parameters as described by Hassan et al. (2015).

### Stress Susceptibility Index

The measure of resistance grounded on minimal yield loss under any stress in comparison to optimal environments is termed as SSI. It is used to illustrate the relative tolerance level for any stress in varieties by using the formula as described by Fischer and Maurer (Fischer and Maurer, 1978).

### Stress Tolerance Index

The tool for determination of the high yield and stress tolerance potential of genotypes is termed as STI. Different growth parameters for STI were calculated by using following the formulae explained by Fernandez (1992) and Amin et al. (2014).

### Biochemical Analysis

After morpho-physiological analysis, the genotypes were screened on the basis of variance analysis, SSI, and STI; the three water stress-tolerant and two water stress-susceptible genotypes, i.e., five genotypes, were finally selected for the biochemical analysis. The fourth true leaf of each genotype’s replicates was collected for biochemical analysis. This analysis was performed on leaf samples for all treatments according to the following methods.

The extraction of the antioxidant enzyme was performed by taking 0.1 *g* of fresh leaf sample from each cotton genotype, then grinding it in 1 ml of 50 mM cold phosphate buffer having a pH of 7.8. It was centrifuged at 14,000 rpm for 10 min. The resultant supernatant was utilized for enzyme activity determination. For soluble protein estimation in leaf sample, 5 μl of extracted supernatant and 95 μl of 0.1 N sodium chloride were mixed with 1 ml of dye reagent. For dye preparation, 0.02 *g* of Coomassie Brilliant Blue G-250 dye was dissolved in 10 ml of 95% ethanol and 20 ml phosphoric acid and diluted to 200 ml. The mixture was left for 5 min to allow the formation of the protein dye complex. At 595 nm, the absorbance was measured.

The superdioxide dismutase (SOD) activity estimation was done from cotton leaves emulsified in a medium composed of 0.1 mM EDTA, 50 mM potassium phosphate buffer, and 1 mM dithiothreitol (Dixit et al., 2001). The SOD activity was estimated by measuring its ability in preventing the photochemical reduction of nitroblue tetrazolium (Giannopolitis and Ries, 1977).

For peroxidase (POD) and catalase (CAT) estimation, cotton leaves were homogenized in a medium composed of 50 mM potassium phosphate buffer, 0.1 mM EDTA, and 1 mM DTT. POD activity was quantified by using the method described by Chance and Maehly (Chance and Maehly, 1955). Similarly, the ascorbate peroxidase (APX) activity was estimated using Erel’s method (Erel, 2004).

For estimation of protease activity, the leaf sample was homogenized in a medium composed of 50 mM potassium phosphate buffer. Its activity was determined by the casein digestion assay (Drapeau, 1976). By this method, one unit is the amount of enzyme that releases acid soluble fragments equivalent to 0.001 at an absorbance of A_280_ per minute at 37°C and pH 7.8. Enzyme activity was expressed on a fresh weight basis.

Lipid peroxidation level in the leaf tissue is measured in terms of malondialdehyde (MDA), which is a product of lipid peroxidation by the thiobarbituric acid reaction utilizing the Heath and Packer method (Heath and Packer, 1968) with slight changes as described by Dhindsa et al. (1981) and Zhang and Kirkham (1994). After centrifuging at 10,000 rpm for 10 min, the absorbance at 532 nm was taken. Then, the value of the non-specific absorption taken at 600 nm was subtracted. By using an extinction coefficient of 155 mM^–1^ cm^–1^, MDA concentration was determined.

Total oxidant status (TOS) was calculated by the method mentioned by Harma et al. (2005). Assay assortment had reagent R_1_, which is a stock solution containing 0.38 *g* of xylenol orange in 500 μl of 25 mM H_2_SO_4_, 0.4 *g* of NaCl, 500 μl of glycerol, and volumed up to 50 ml with 25 mM H_2_SO_4_, and reagent R_2_ contains 0.0317 *g* of o-dianisidine, 0.0196 *g* of ferrous ammonium sulfate (II) and sample extract. After 5 min of adding sample extract, the absorption at 560 nm was measured by a spectrophotometer (HITACHI-2800).

The amount of chlorophyll (a and b) and carotenoids were calculated by the method of Arnon (1949) in which 0.2 *g* of the leaf was ground in 80% acetone extract at −4°C. It was then centrifuged at the speed of 10,000 *g* for 5 min. At 645, 663, and 480 nm, the absorbance of the supernatant was measured. Formulae used were as follows:

Chla(mg/gf.wt.)=[12.7⁢(OD⁢ 663)-2.69⁢(OD⁢ 645)×V/1,000×W]

Chlb(mg/gf.wt.)=[22.9⁢(OD⁢ 645)-2.69⁢(OD⁢ 663)×V/1,000×W]

Carotenoids(mg/gf.wt.)=[Acar/EM]× 1,000Acar=OD 480+ 0.114(OD 663)-638(OD 645)

V=sample⁢volume,W=weight⁢of⁢tissues,Em= 2,500.

Esterase activity was determined by the method described by van Asperen (1962) with some changes. Leaf sample of 500 mg was homogenized in 5 ml of phosphate buffer, containing 1 mM of EDTA, 1 mM of PMSF, 1 mM of PTU, and 20% glycerol, by using mini bead beater and centrifuging at 10,000 rpm for 20 min at 4°C. The enzyme estimation was done by the supernatant obtained. Different standards (0.1–0.9 μM interval) of esterase were prepared from stock solutions in 1,000 μl of distilled water. To all the standard solutions and blank, 5 ml of phosphate buffer was added; 1 ml of staining solution was added and placed at 30°C in dark for 20 min with gentle shaking (incubation). Then, absorbance at 590 nm was measured. Standard curves were prepared by the use of standards.

Micro colorimetric technique (Ainsworth and Gillespie, 2007) was used for the total phenolic assay, which utilizes Folin–Ciocalteu (F-C) reagent. A standard curve was prepared using different concentrations of gallic acid, and a linear regression equation was calculated. Phenolic content (gallic acid equivalents) of samples was determined by using the linear regression equation.

The total flavonoid content was determined according to the aluminum chloride colorimetric method (Ainsworth and Gillespie, 2007). The total antioxidant capacity of serum samples was verified by standardizing the Erel method (Erel, 2004) and making necessary modifications.

### Statistical Analysis

The screening was carried out in quadruplicate using CRD. Variance analysis was established for significance estimation (*p* < 0.05). Moreover, principal component analysis, agglomerative hierarchical clustering, and simple correlation coefficients were estimated by using the computer software Microsoft Excel along with XLSTAT version 2012.1.02., copyright Addinsoft 1995–2012^1^. Nevertheless, SSIs and STIs were calculated using the formulae given by Fischer and Maurer (1978); Fernandez (1992), and Amin et al. (2014), respectively.

## Results

### Morpho-Physiological Traits

Genotypic differences for all the morphological and physiological traits revealed highly significant differences except (TR, 0.059^ns^), and two water regimes also divulged the same pattern as indicated in Table 3. Moreover, Table 3 also shows that the interaction between them (G × W) was highly significant for root length (RL, 5.163), shoot length (SL, 11.751), excised leaf water loss (ELWL, 0.041), and stomatal conductance (SC, 7.406), and significant for relative water content (RWC, 12.961), and photosynthesis (PS, 1.9) whereas non-significant for (TR, 0.003). The minimum and maximum values for RL, SL, RWC, ELWL, SC, TR, and PS under normal and drought conditions are presented in Table 4.

**TABLE 3:** Mean squares values of variance analysis for different traits.

Source of variation	Df	RL	SL	RWC	ELWL	SC	TR	PS
Genotypes (G)	22	13.308**	37.415**	162.391**	0.469**	395.5**	0.059ns	317.6**
Water regimes (W)	1	399.500**	5210.8**	3297.3**	5.162**	399.1**	0.202ns	73.2**
G × W	22	5.163*	11.751**	12.961*	0.041**	7.406**	0.003ns	1.9*
Error	92	0.849	1.031	6.447	0.031	0.364	0.00	1.0
Total	137	CV: 5.10%	CV:3.75%	CV:3.43%	CV:10.76%	CV:4.57%	CV:5.65%	CV:5.18%

*Significat at 5%.**Highly Significant at 1%.^*ns*^Non-significant.RL, Root Length; SL, Shoot Length; RWC, Relative Water Content; ELWL, Excised Leaf Water Loss; SC, Stomatal Conductance; TR, Transpiration rate; PS, Photosynthesis; and Df, Degree of freedom.

**TABLE 4:** Stress susceptibility and stress tolerance indices for morpho-physiological traits.

Genotype	RL		SL		RWC		ELWL		SC		TR		PS	
	SSI	STI	SSI	STI	SSI	STI	SSI	STI	SSI	STI	SSI	STI	SSI	STI
NIAB-135	0.41	0.93	0.71	0.74	0.72	0.91	0.73	0.77	0.12	0.97	0.13	0.96	0.10	0.99
SLH-375	0.41	0.93	0.70	0.74	0.85	0.89	0.98	0.69	0.19	0.96	0.21	0.94	0.20	0.99
CIM-554	0.51	0.91	1.09	0.60	1.11	0.86	0.41	0.87	0.29	0.93	0.29	0.92	0.26	0.98
NIAB-846	0.95	0.84	0.91	0.66	0.79	0.90	1.27	0.60	0.37	0.91	0.33	0.91	0.13	0.99
BH-160	0.43	0.93	1.26	0.53	1.34	0.83	1.02	0.68	0.66	0.85	0.62	0.83	0.39	0.97
CIM-663	1.11	0.81	0.77	0.72	0.85	0.89	0.47	0.85	0.75	0.83	0.87	0.76	1.25	0.91
FH-Lalazar	1.30	0.78	1.14	0.58	1.11	0.86	1.24	0.61	0.77	0.82	0.71	0.80	0.35	0.98
BH-199	0.94	0.84	0.99	0.63	0.77	0.90	0.91	0.72	0.82	0.81	0.76	0.79	0.42	0.97
NIAB-852	1.75	0.70	1.33	0.51	1.26	0.84	1.44	0.55	0.87	0.80	0.78	0.78	0.35	0.98
NIAB-512	0.61	0.90	1.06	0.61	0.46	0.94	0.59	0.81	1.11	0.75	1.17	0.67	1.41	0.90
NIAB-112	0.65	0.89	1.06	0.61	0.87	0.89	0.83	0.74	1.10	0.75	0.97	0.73	0.37	0.97
Gomal-105	1.10	0.81	1.06	0.61	1.28	0.84	1.01	0.68	1.12	0.75	1.17	0.67	1.38	0.90
FH-498	0.55	0.91	0.84	0.69	0.76	0.91	0.98	0.69	1.12	0.74	1.10	0.69	1.08	0.92
MARVI	1.14	0.80	1.13	0.58	1.43	0.82	1.25	0.61	1.16	0.74	1.20	0.66	1.41	0.90
MNH-786	1.09	0.81	0.84	0.69	1.09	0.87	0.50	0.84	1.15	0.74	1.20	0.66	1.44	0.90
FH-326	1.03	0.82	0.87	0.68	0.94	0.88	0.90	0.72	1.25	0.71	1.27	0.64	1.41	0.90
FH-142	1.46	0.75	0.89	0.67	1.10	0.86	1.09	0.66	1.35	0.69	1.30	0.64	1.14	0.92
CIM-496	0.59	0.90	1.07	0.61	1.34	0.83	1.26	0.61	1.35	0.69	1.37	0.61	1.64	0.88
NIBGE-2	1.96	0.66	1.09	0.60	0.94	0.88	1.03	0.68	1.39	0.68	1.39	0.61	1.49	0.89
FH-490	0.78	0.87	0.94	0.65	0.77	0.90	1.03	0.68	1.55	0.65	1.29	0.64	0.18	0.99
CIM-343	1.03	0.82	0.95	0.65	0.84	0.90	0.95	0.70	1.55	0.65	1.65	0.54	2.34	0.83
VH-363	0.89	0.85	0.91	0.66	0.75	0.91	1.18	0.63	1.56	0.64	1.59	0.55	1.95	0.86
BH-167	1.93	0.67	1.28	0.53	1.50	0.81	1.26	0.61	1.67	0.62	1.61	0.55	1.63	0.88

***Legends:** SSI, Stress susceptibility index; STI, Stress tolerance index; RL, Root length; SL, Shoot length; RWC, Relative water content; ELWL, Excised leaf water loss; SC, Stomatal conductance; TR, Transpiration rate; and PS: Photosynthesis.*

The SSI and STI were also computed. On the basis of SSI and STI regarding percentage decrease in RL under drought conditions in comparison to normal conditions, we categorized the accessions into three groups. The genotype having the least SSI value and least percentage decrease is the most tolerant genotype. NIAB-135 (0.41), SLH-375 (0.41), BH-160 (0.43), CIM-554 (0.51), and FH-498 (0.55) were categorized as most tolerant genotypes in decreasing order, whereas FH-490 (0.78), VH-363 (0.89), BH-199 (0.94), and NIAB-846 (0.95) fall in medium category, and the most susceptible accessions were FH-142 (1.46), NIAB-852 (1.75), BH-167 (1.93), and NIBGE-2 (1.96) in the increasing order (Table 4).

The STI and SSI were estimated on the basis of their percentage decrease in SL, and all the accessions were categorized into three groups. The accessions/varieties, namely, SLH-375 (SSI: 0.70 and STI: 0.74), NIAB-135 (SSI: 0.71 and STI: 0.74), CIM-663 (SSI: 0.77 and STI: 0.72), and FH-498 (SSI: 0.84 and STI: 0.69) were found to be the most tolerant under drought stress in decreasing order, whereas VH-363 (SSI: 0.91 and STI: 0.66), FH-490 (SSI: 0.94 and STI: 0.65), CIM-343 (SSI: 0.95 and STI: 0.65), and BH-199 (SSI: 0.99 and STI: 0.63) were categorized in medium category, and the accessions BH-160 (SSI: 1.26 and STI: 0.53), BH-167 (SSI: 1.28 and STI: 0.53), and NIAB-852 (SSI: 1.33 and STI: 0.51) were the most susceptible in increasing order (Table 4).

The accessions were categorized on the basis of SSI and STI for RWC. The most tolerant were NIAB-512 (SSI: 0.46 and STI: 0.94), NIAB-135 (SSI: 0.72 and STI: 0.91), VH-363 (SSI: 0.75 and STI: 0.91), and FH-498 (SSI: 0.76 and STI: 0.91) in decreasing order. The accessions that fall in medium category were CIM-663 (SSI: 0.85 and STI: 0.89), SLH-375 (SSI: 0.85 and STI: 0.89), NIAB-112 (SSI: 0.87 and STI: 0.89), and FH-326 (SSI: 0.94 and STI: 0.88). The most susceptible accessions in increasing order were BH-160 (SSI: 1.34 and STI: 0.83), MARVI (SSI: 1.43 and STI: 0.82), and BH-167 (SSI: 1.50 and STI: 0.81; Table 4).

The accessions were categorized into three groups for ELWL. The most tolerant were CIM-554 (SSI: 0.41 and STI: 0.87), CIM-663 (SSI: 0.47 and STI: 0.85), and MNH-786 (SSI: 0.50 and STI: 0.84). NIAB-512 (SSI: 0.59 and STI: 0.81), BH-199 (SSI: 0.91 and STI: 0.72), CIM-343 (SSI: 0.95 and STI: 0.70), and FH-498 (SSI: 0.98 and STI: 0.69) were in medium category. The most susceptible accessions were BH-167 (SSI: 1.26 and STI: 0.61), NIAB-846 (SSI: 1.27 and STI: 0.60), and NIAB-852 (SSI: 1.44 and STI: 0.55) in decreasing order (Table 4).

The accessions were categorized into three groups on the basis of percent decrease in drought conditions, SSI, and STI values for SC. NIAB-135 (SSI: 0.12 and STI: 0.97), SLH-375 (SSI: 0.19 and STI: 0.96), CIM-554 (SSI: 0.29 and STI: 0.93), and NIAB-846 (SSI: 0.37 and STI: 0.91) were identified as the most tolerant varieties, and CIM-663 (SSI: 0.75 and STI: 0.83), BH-199 (SSI: 0.82 and STI: 0.81), and NIAB-852 (SSI: 0.87 and STI: 0.80) fall in medium category in decreasing order, respectively. The most susceptible genotypes were BH-167 (SSI: 1.67 and STI: 0.62), VH-363 (SSI: 1.56 and STI: 0.64), and CIM-343 (SSI: 1.55 and STI: 0.65) in decreasing order, respectively, (Table 4).

Similarly, the accessions were categorized in three groups on the basis of percent decrease in drought conditions and SSI and STI values for transpiration rate. The accession, namely, NIAB-135 (SSI: 0.13 and STI: 0.96), SLH-375 (SSI: 0.21 and STI: 0.94), CIM-554 (SSI: 0.29 and STI: 0.92), and NIAB-846 (SSI: 0.33 and STI: 0.91) fall in tolerant category in decreasing order, and FH-Lalazar (SSI: 0.71 and STI: 0.80), BH-199 (SSI: 0.76 and STI: 0.79), NIAB-852 (SSI: 0.78 and STI: 0.78), CIM-663 (SSI: 0.87 and STI: 0.76), and NIAB-112 (SSI: 0.97 and STI: 0.73) were in medium category. Whereas, VH-363 (SSI: 1.59 and STI: 0.55), BH-167 (SSI: 1.61 and STI: 0.55), and CIM-343 (SSI: 1.65 and STI: 0.54) were the most susceptible accessions in increasing order (Table 4).

We categorized all the accession in three groups on the basis of percent decrease in drought conditions and SSI and STI values for photosynthesis. NIAB-135 (SSI: 0.10 and STI: 0.99), FH-490 (SSI: 0.18 and STI: 0.99), SLH-375 (SSI: 0.20 and STI: 0.99), and CIM-554 (SSI: 0.26 and STI: 0.98) fall in tolerant category in decreasing order. NIAB-852 (SSI: 0.35 and STI: 0.98), FH-Lalazar (SSI: 0.35 and STI: 0.98), NIAB-112 (SSI: 0.37 and STI: 0.97), BH-160 (SSI: 0.39 and STI: 0.97), and BH-199 (SSI: 0.42 and STI: 0.97) were in medium category, whereas BH-167 (SSI: 1.63 and STI: 0.88), VH-363 (SSI: 1.95 and STI: 0.86), and CIM-343 (SSI: 2.34 and STI: 0.83) were the most susceptible accessions in increasing order (Table 4).

#### Correlation Among Morpho-Physiological Traits

A positively strong correlation was observed in SC and TR under normal conditions (0.554). A negatively strong correlation was found in PS and SC (−0.504) as shown in Table 5. Under drought conditions, a positively strong correlation was found in TR with RWC (0.510) and SC (0.584) and RWC with PS (0.452). A negative correlation was found in SC and SL (−0.428) and SC and PS (−0.446; Table 6).

**TABLE 5:** Principle component analysis of different morpho-physiological traits in cotton under normal and drought conditions.

	Control conditions				Drought conditions		
	PC1	PC2	PC3	PC4	PC1	PC2	PC3
Eigenvalue	1.98	1.62	1.21	1.00	2.19	1.88	1.07
Total variance (%)	28.32	23.20	17.34	14.22	31.31	26.85	15.25
Cumulative variance (%)	28.32	51.52	68.86	83.09	31.31	58.16	73.41

**TABLE 6:** Correlation matrix for morpho-physiological traits under normal and drought conditions.

Normal conditions	Variables	RL-N	SL-N	RWC-N	ELWL-N	SC-N	TR-N	PS-N
	RL-N	**1**						
	SL-N	0.009	**1**					
	RWC-N	0.250	0.058	**1**				
	ELWL-N	0.174	−0.234	−0.139	**1**			
	SC-N	0.173	−0.255	−0.035	0.134	**1**		
	TR-N	0.189	−0.346	0.251	0.166	**0.554**	**1**	
	PS-N	0.035	−0.202	0.326	0.063	−**0.504**	0.287	**1**

**Drought conditions**	**Variables**	**RL-D**	**SL-D**	**RWC-D**	**ELWL-D**	**SC-D**	**TR-D**	**PS-D**

	RL-D	**1**						
	SL-D	0.202	**1**					
	RWC-D	0.381	0.039	**1**				
	ELWL-D	0.068	0.076	−0.064	**1**			
	SC-D	−0.065	−**0.428**	0.024	−0.052	**1**		
	TR-D	0.225	−0.095	**0.510**	0.161	**0.584**	**1**	
	PS-D	0.276	0.269	**0.452**	0.285	−**0.446**	0.332	**1**

*Values in bold are different from 0 with a significance level alpha = 0.05. **Legends:** RL, Root Length; SL, Shoot Length; RWC, Relative Water Content; SC, Stomatal Conductance; TR, Transpiration Rate; PS, Photosynthesis; N, Normal conditions; and D, Drought conditions.*

#### Principal Component Analysis for Morpho-Physiological Traits

**FIGURE 1:**
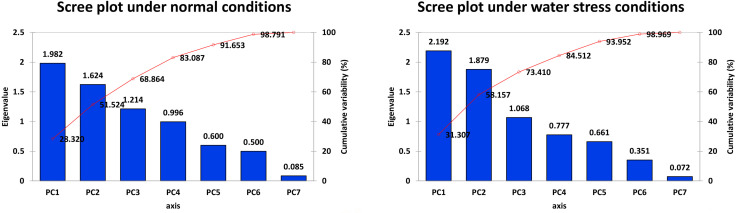
Scree plot between eigenvalues and factors under normal and drought conditions.

It is evident from the scree plot that in this study, seven factors altogether contribute to the total variation under normal and water-stressed conditions. However, seven among the four principal components (PCs) and three PCs divulged eigen values ≥ 1 under both conditions. Four PCs under normal and three PCs under water-stressed interpolated cumulative variance of 83.09 and 73.41%, respectively, among the cotton genotypes evaluated for drought related traits (Figure 1 and Table 5). Table 5 illustrates that rest of the components revealed only 16.91 and 26.59% of the total variation under normal and water-stressed conditions, respectively. The PC1 showed the maximum variability of 28.32 and 31.31% shadowed by PC 2, 23.20 and 26.85%, and PC 3, 17.34 and 15.25%, under normal and water-stressed conditions, respectively.

Characters like TR-N, SC-N, SL-N, PS-D, RWC-D, and RL-D showed substantial positive contribution in PC 1 in a range from 1.084 (PS-N) to 36.23 (TR-N) and from (SC-D) 1.25 to (PS-D) 29.590, under both conditions. Whereas, PC 2 was associated with diversity among genotypes due to PS-N (48.775), RWC-N (24.616), SC-N (23.615), SC-D (45.286), TR-D (26.477), and SL-D (19.790) with their positive contribution under normal and water-stressed conditions, respectively. The PC 3 elucidated variation among cotton genotypes owing to SL-N (25.808), RWC-N (23.218), RL-N (19.559), ELWL-D (75.944), RWC-D (11.527), and RL-D (7.266) with positive influence for both conditions. The PC4 explicated the variance among the genotypes for ELWL-N (43.892) and RL-N (37.751) with positive denominations under normal conditions (Table 7).

**TABLE 7:** Contribution of the variables (%) under normal and drought conditions.

		PC1	PC2	PC3	PC4
Normal conditions	RL-N	9.051	1.226	19.559	37.751
	SL-N	17.473	0.003	25.808	4.668
	RWC-N	3.281	24.616	23.218	1.456
	ELWL-N	9.103	0.707	16.560	43.892
	SC-N	23.386	23.615	5.832	4.065
	TR-N	36.623	1.058	0.228	8.086
	PS-N	1.084	48.775	8.796	0.082
Drought conditions	RL-D	17.738	0.005	7.266	
	SL-D	5.318	19.790	0.585	
	RWC-D	25.819	3.709	11.527	
	ELWL-D	4.035	0.433	75.944	
	SC-D	1.205	45.286	0.571	
	TR-D	16.295	26.477	1.737	
	PS-D	29.590	4.300	2.370	

***Legends:** RL, Root Length; SL, Shoot Length; RWC, Relative Water Content; SC, Stomatal Conductance; TR, Transpiration Rate; PS, Photosynthesis; N, Normal conditions; and D, Drought conditions.*

**FIGURE 2:**
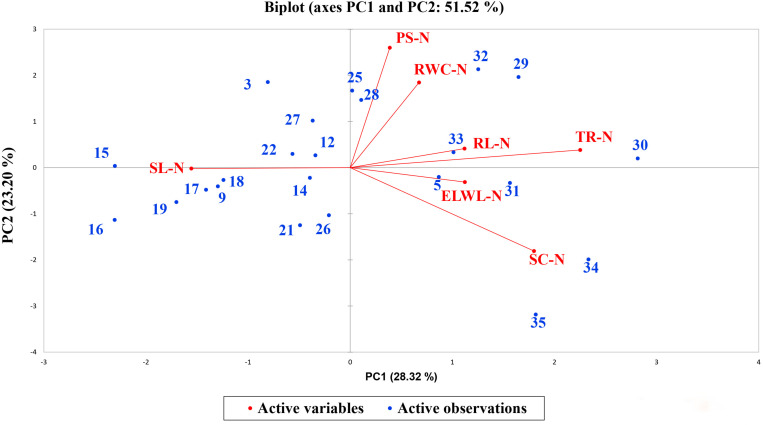
Biplot illustrating contribution of various morpho-physiological traits under normal conditions.

**FIGURE 3:**
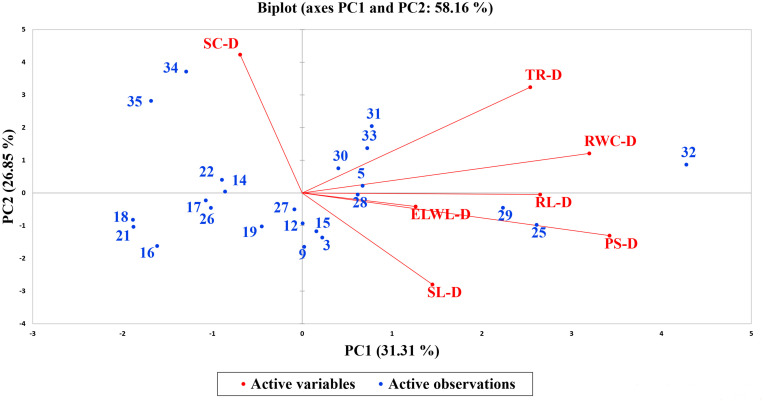
Biplot illustrating contribution of various traits under drought conditions.

A biplot between PC 1 and PC 2 in Figures 2, 3 divulged that traits and genotypes are superjacent on the plot as vectors under normal and water-stressed conditions. It was evident from the distance of each trait with respect to PC 1 and PC 2 that these traits were responsible for the variation among the genotypes. The biplot revealed that SC-N, TR-N, PS-N, SL-N, RWC-N, SC-D, TR-D, RWC-D, RL-D, ELWL-D, PS-D, and SL-D altogether are instrumental in the variability in cotton germplasms under study (Figures 2, 3). Moreover, PC 1 and PC 2 were responsible for 51.52 and 58.16% variation among the genotypes under normal and water-stressed conditions, respectively.

#### Cluster Analysis

Morpho-physiological data was collected for all the accessions, and they were subjected to variance analysis, which showed statistically significant results among all the varieties. Agglomerative hierarchical clustering clustered all accessions on the similarity basis of SSI values into five diverse classes. Within the class, 43.09% of the variation was observed and 56.91% variation was observed between the classes.

**FIGURE 4:**
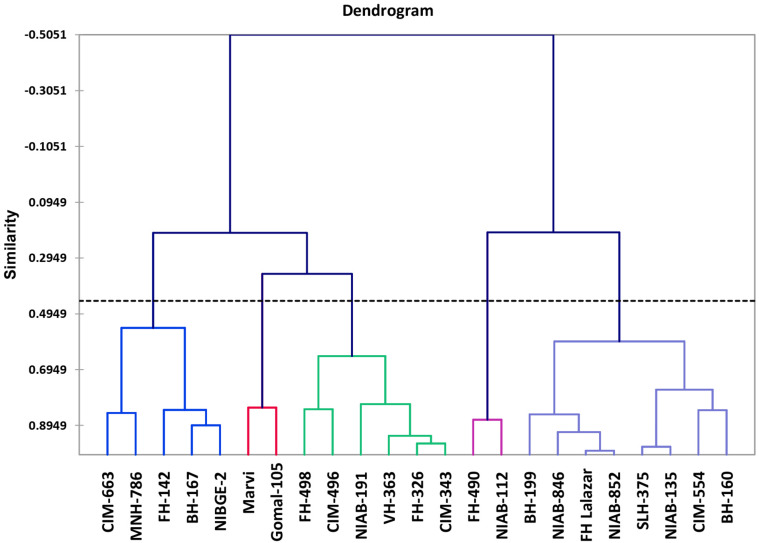
Dendrogram of cotton genotypes based on stress susceptibility index (SSI).

Class 1 showed six genotypes, which were moderately drought tolerant: FH-498, CIM-496, NIAB-512, VH-363, FH-326, and CIM-343. Class 2 showed drought tolerant varieties, which include NIAB-112 and FH-490. Class 3 showed highly tolerant varieties including BH-160, CIM-554, NIAB-135, SLH-375, NIAB-852, FH-Lalazar, NIAB-846, and BH-199. Class 4 contains drought-susceptible varieties: NIBGE-2, BH-167, FH-142, MNH-786, and CIM-663. Class 5 contains the most susceptible genotypes: MARVI and Gomal-105 (Figure 4).

### Biochemical Results

#### Lycopene and Beta-Carotene

**FIGURE 5:**
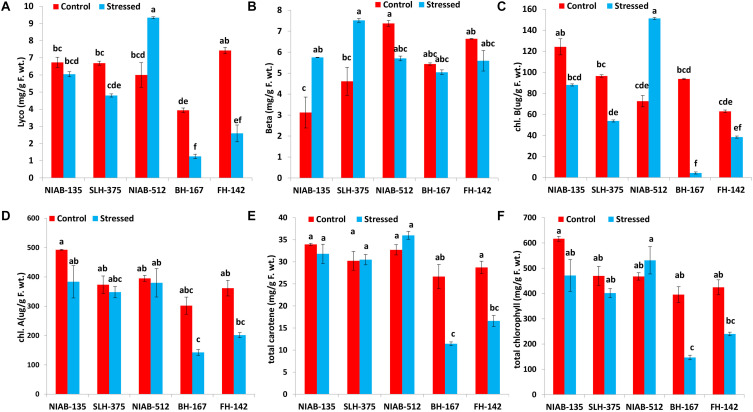
Graphical representation of lycopene **(A)**, beta-carotene **(B)**, Chl b **(C)**, Chl a **(D)**, total carotene **(E)**, and total chlorophyll content **(F)** in cotton under normal and drought conditions. Different lowercase letters indicate statistical analysis, i.e., Tukey’s honestly significant difference test or Tukey’s HSD.

Generally, lycopene content (mg/g F. wt.) was decreased under drought stress in all tolerant and sensitive genotypes except NIAB-512 (5.997–9.340) in which a significant increase under stress was observed. Lycopene content was significantly decreased under drought stress in relatively tolerant genotype, i.e., NIAB-135 (6.731–6.044). However, lycopene content was significantly decreased in SLH-375 (6.688–4.803), BH-167 (3.938–1.252), and FH-142 (7.425–2.594) with the relatively higher drop in sensitive genotypes. The most sensitive genotype BH-167 also maintained the lowest lycopene content under drought and non-stress conditions (Figure 5A). Whereas, generally, beta-carotene content in all genotypes under stressed conditions was more than the non-stressed conditions. Beta-carotene content (mg/g F. wt.) in tolerant genotypes was significantly increased under drought stress, i.e., NIAB-135 (3.122–5.747) and SLH-375 (4.611–7.516). The susceptible genotypes showed a non-significant decrease in beta-carotene content under drought stress in comparison to non-stress conditions, i.e., BH-167 (5.433–5.037) and FH-142 (6.634–5.588). The most susceptible genotype, however, maintained the lowest value of beta-carotene content under stressed conditions (Figure 5B).

#### Chlorophyll A and Chlorophyll B

Chlorophyll a content (μg/g F. wt.) was reduced under drought stress in all varieties, but the decrease was not significantly different. The most tolerant genotype, i.e., NIAB-135, maintained higher chlorophyll a content (492.459–383.362) than all other genotypes, whereas the most susceptible genotype, i.e., BH-167, contains the least chlorophyll a content (142.439 ± 9.78) under drought stress (Figure 5D).

Similarly, chlorophyll b content (μg/g F. wt.) was decreased in all genotypes under drought stress except NIAB-512, which showed a significant increase from 72.568 to 151.234 under stress condition. All genotypes showed significantly different results under stressed conditions in comparison to the non-stress conditions except for NIAB-135, which showed non-significant differences. All the varieties excluding NIAB-512 followed the pattern of simultaneous decrease in chlorophyll b content from the most tolerant toward the most susceptible genotype. The most susceptible genotype showed the lowest chlorophyll b content under drought stress (Figure 5C).

#### Total Carotene and Total Chlorophyll

The total carotene content (mg/g F. wt.) was decreased in all genotypes including tolerant and sensitive except NIAB-512 in which anomaly was observed; the total carotene content increased non-significantly (32.724–35.997). Sensitive genotypes showed a significant decrease in the total carotene content, i.e., BH-167 (26.653–11.425) and FH-142 (28.735–16.611). However, in tolerant genotypes, the decrease was non-significant. The most sensitive genotype maintained the lowest value of total carotene content under drought stress and control conditions (Figure 5E).

All the genotypes generally showed statistically a significant increase or decrease in total chlorophyll content (mg/g F. wt.) except the most sensitive genotype, i.e., BH-167. The general trend of decrease in total chlorophyll in all the genotypes under stressed conditions was observed except in NIAB-512, which exhibited the non-significant increase in total chlorophyll content (467.592–531.316) under water stress (Figure 5F).

#### Total Soluble Proteins and Total Phenolic Contents

**FIGURE 6:**
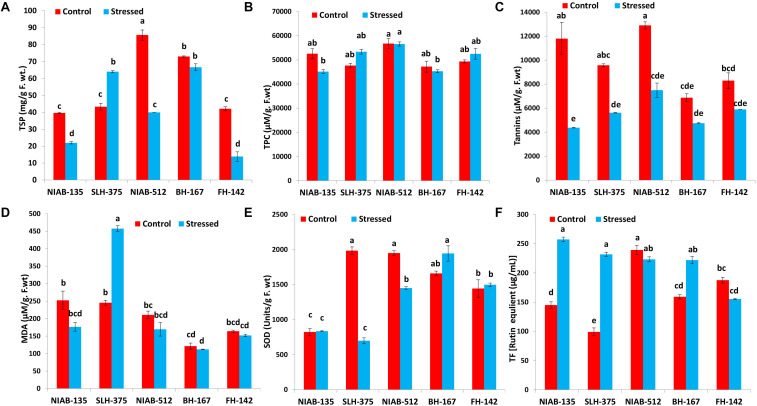
Graphical representation of TSP **(A)**, TPC **(B)**, Tannis **(C)**, MDA **(D)**, SOD **(E)**, and TF **(F)** of cotton under normal and drought conditions. Different lowercase letters indicate statistical analysis, i.e., Tukey’s honestly significant difference test or Tukey’s HSD.

On the whole, all the genotypes showed a statistically significant increase or decrease in total soluble protein (mg/g F. wt.) except the most sensitive genotype, i.e., BH-167 (73.0–66.667). The general trend of decrease in total soluble proteins in all the genotypes under stressed conditions was observed except NIAB-512, which exhibited a significant increase (85.667–40.0) under water stress (Figure 6A).

All together, no significant decrease or increase in any genotype was observed for total phenolic contents (μM/g. F.wt). The least tolerant genotype like SLH-375 showed a significant increase (47,625–53,350), while the least susceptible genotype, i.e., NIAB-512, expressed a non-significant decrease (56,750–56,575) in total phenolic content under drought stress. However, the most tolerant and most susceptible genotypes [NIAB-135 (52,550–45,150) and BH-167 (47,200–45,200), respectively] showed a significant decrease in the TPC under drought stress. The most susceptible genotype maintained the least value for TPC under control conditions, i.e., BH-167 (Figure 6B).

#### Tannins and Malondialdehyde

Tolerant genotypes showed a significant decrease in tannins content (μM/g. F.wt) under drought stress, i.e., NIAB-135 (11,800–4,375), SLH-375 (9,575–5,617.5), and NIAB-512 (12,900–7,500). However, the susceptible genotypes showed non-significant decrease under drought stress, i.e., BH-167 and FH-142 (8,300–5,900; Figure 6C).

Overall, no significant increase or decrease in MDA content (μM/g. F.wt) was observed in either of the genotypes except SLH-375 (245.419–457.484) in which a significant increase in the MDA content was observed under drought stress. The tolerant genotype (i.e., SLH-375) showed the highest value of MDA content under drought stress. The most susceptible genotype showed the lowest values of MDA content (121.548–112.258) under normal and drought stress conditions (Figure 6D).

#### Superoxide Dismutase and Total Flavonoids

No general trend was observed in the SOD content (units/g F. wt) and in total flavonoids [rutin equivalent (μg/ml)] in either of the genotypes. Some genotypes showed a non-significant increase in SOD content under drought stress, while others showed a significant decrease in the content under drought stress. The tolerant genotype (i.e., SLH-375) showed the highest value of SOD content (1,984.569 ± 53.226) under control conditions, and at the same time, the lowest value observed for SOD content (698.976 ± 40.452) was also of SLH-375 under drought stress (Figure 6E).

The tolerant genotypes showed a significant increase in the total flavonoid content [rutin equivalent (μg/ml)] under stress conditions. The highest value recorded for total flavonoid content was of genotype NIAB-135 (257.457 ± 3.992) under drought stress, and the lowest value (99.132 ± 6.803) observed was for SLH-375 under control conditions (Figure 6F).

#### Total Oxidant Status and Ascorbic Acid

**FIGURE 7:**
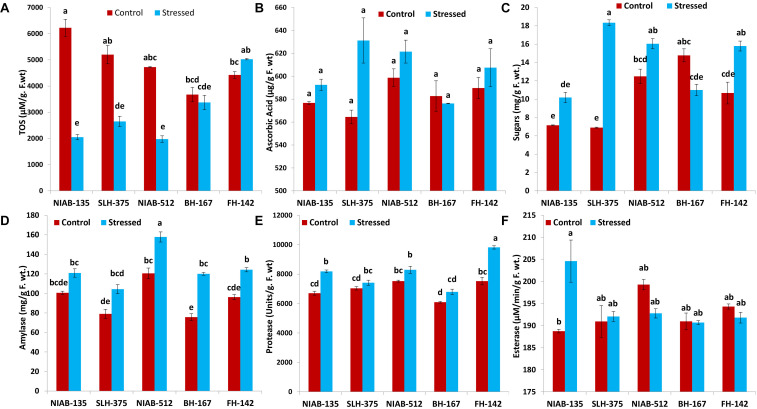
Graphical representations of total oxidant status (TOS; **A)**, ascorbic acid **(B)**, sugars **(C)**, amylase **(D)**, protease **(E)**, and esterase **(F)** of cotton genotypes under non-stressed and drought stressed conditions. Different lowercase letters indicate statistical analysis, i.e., Tukey’s honestly significant difference test or Tukey’s HSD.

All the tolerant genotypes showed a significant decrease in the (TOS, μM/g F.wt), whereas the susceptible genotypes (i.e., FH-142; 4,425–5,027 and BH-167; 3,675–3,375) showed non-significant increase and decrease, respectively. The most tolerant genotype showed the highest value for TOS content under control conditions, i.e., NIAB-135 (6,225 ± 325). The lowest value (1,975 ± 25) for TOS content was observed in NIAB-512 under drought stress (Figure 7A).

A non-significant increase in ascorbic acid content (μg/g F. wt) under drought stress was observed in all genotypes except BH-167, which showed a non-significant decrease (582.750–576.250) under drought stress. Ascorbic acid content observed in all the genotypes showed insignificant differences. The tolerant genotype SLH-375 showed the highest value (631.250 ± 19.750) among all genotypes under drought stress, and the lowest value was also observed in SLH-375 under control conditions (Figure 7B).

#### Sugars and Amylase

An increase in sugar content (mg/g F. wt.) was observed in all genotypes except the most susceptible genotype, i.e., BH-167 (14.783–11.000). SLH-375 showed a significant increase in sugar content (6.903–18.340) under drought stress. FH-142 also showed a significant increase in sugar content (10.672–15.800) under drought stress. The remaining genotypes showed non-significant differences (Figure 7C).

The tolerant genotypes showed non-significant differences under control and drought stress. The remaining genotypes showed significant increase under drought stress. Overall, the amylase content (mg/g F. wt.) in all genotypes was increased under drought stress. However, the increase was significant in some genotypes and non-significant in others. The highest values (12.566 ± 3.585) of amylase were observed in NIAB-512 in control as well drought stress conditions (Figure 7D).

#### Protease and Esterase

Overall, the genotypes showed a non-significant increase in the protease activity (units/g F. wt) under drought stress except of SLH-375 and FH-142, which showed a significant increase (7,035–7,415 and 7,525–9,820, respectively) under drought stress. The highest value (9,820 ± 110) for protease content was observed in FH-142 under drought stress, whereas the lowest value (6,095 ± 65) was observed in BH-167 under control conditions (Figure 7E).

In general, the non-significant decrease was observed in all the genotypes under drought stress for the esterase activity (μM/min/g F. wt) except the most tolerant genotype, i.e., NIAB-135, in which a significant increase in esterase content (188.854–204.621) was observed under drought stress conditions. The highest observed value (204.621 ± 4.764) for esterase content was observed in NIAB-135 under drought stress conditions, while the lowest value was also observed in the same genotype under control conditions (Figure 7F).

#### Proline and CAT

**FIGURE 8:**
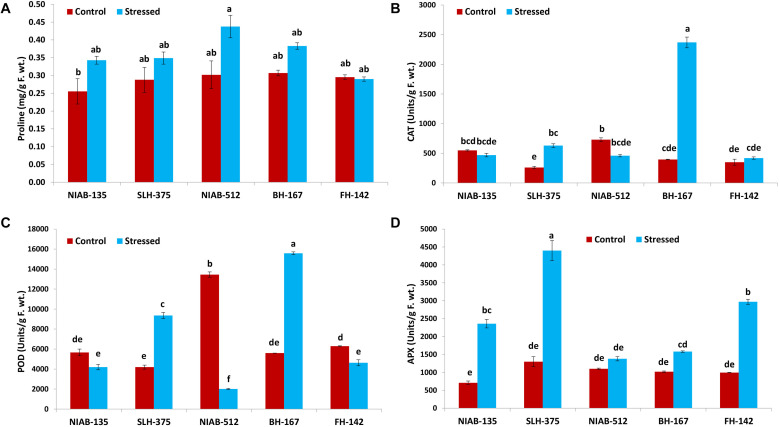
Graphical representation of proline **(A)**, catalase (CAT; **B)**, peroxidase (POD; **C)**, and ascorbate peroxidase (APX; **D)** of cotton genotypes under non-stressed and drought-stressed conditions. Different lowercase letters indicate statistical analysis, i.e., Tukey’s honestly significant difference test or Tukey’s HSD.

Overall, no significant differences were observed among all the genotypes. The highest value (0.438 ± 0.032) for proline content (mg/g F. wt.) was observed in NIAB-512 under drought stress conditions, and the lowest (0.256 ± 0.036) was observed in NIAB-135 under control conditions. The value of proline content under drought stress was non-significantly increased except for FH-142 in which the value was decreased non-significantly (Figure 8A).

The CAT activity (units/g F. wt) was significantly increased in some genotypes whereas it decreased non-significantly in others. In the most susceptible genotype, the CAT content increased significantly under drought stress conditions, i.e., BH-167 (395–2,370). In the most tolerant genotype, the CAT content was decreased non-significantly under drought stress conditions (Figure 8B).

#### POD and APX

All the genotypes showed significant differences under drought stress conditions in POD (units/g F. wt) except the most tolerant genotype, which showed non-significant differences, i.e., NIAB-135 (5,663–4,195). The most susceptible genotypes showed a significant increase in the POD content (5,595.0–15,584.9) under drought stress, i.e., BH-167. The tolerant genotype showed a significant increase (4,195–9,358) in POD content under drought stress, i.e., SLH-375. At the same time, the susceptible genotype showed a significant decrease in the POD content (6,295.7–4,626.7) under drought stress, i.e., FH-142. The highest value of POD content was observed in BH-167 (15,584.9 ± 132.7) under drought stress and the lowest value of POD content was observed in NIAB-512 (2,014 ± 49.8) under drought stress conditions (Figure 8C).

All the genotypes, either tolerant or susceptible, showed an increase in APX activity (units/g F. wt) under drought stress. The tolerant genotypes showed a significant increase in APX activity, while the most susceptible genotype, i.e., BH-167, showed vice versa expression under drought stress conditions. The highest value of APX content was observed in SLH-375 (4,400 ± 280) under drought stress, and the lowest value was observed in NIAB-135 (710 ± 50) under control conditions (Figure 8D).

#### Correlation Among Biochemical Traits

A positive and highly strong correlation was found for the following characters under normal conditions: total carotenoids and chlorophyll a (0.925); total chlorophyll and chlorophyll a (0.981); tannins and total carotenoids (0.952); tannins and total phenolic content (0.899); TOS and chlorophyll a (0.951); TOS and total chlorophyll (0.956); TOS and MDA (0.921); ascorbic acid and TF (0.990); amylase and TPC (0.970); esterase and beta-carotene (0.918); CAT and TPC (0.945); POD and TF (0.897); POD and esterase (0.896); and APX and SOD (0.944). A negative and highly strong correlation was found in the following characters under normal conditions: chlorophyll b and beta-carotene (−0.946); proline and chlorophyll a (−0.905); proline and total chlorophyll (−0.951); and proline and TOS (−0.933; Table 8, values below diagonal).

**TABLE 8:** Correlation matrix for biochemical traits under normal and drought conditions.

Variables	Ly	β-Car	Chl a	Chl b	T Car	T Chl	TSP	TPC	Tan	MDA	SOD	TF	TOS	AA	Sug	Amy	Prot	Est	Pro	CAT	POD	APX
Ly	**1**	*0.226*	***0.886***	***0.988***	***0.935***	***0.958***	*−0.198*	*0.543*	*0.617*	*0.143*	*−0.406*	*0.414*	*−0.735*	*0.561*	*0.254*	*0.659*	*0.128*	*0.335*	*0.589*	*−0.631*	*−0.753*	*−0.197*
β-Car	*−*0.062	**1**	*0.517*	*0.104*	*0.501*	*0.395*	*0.296*	*0.421*	*0.104*	***0.991***	*−0.804*	*0.220*	*−0.249*	*0.786*	*0.697*	*−0.473*	*−0.133*	*−0.045*	*−0.140*	*−0.443*	*−0.071*	*0.866*
Chl a	0.562	*−*0.534	**1**	*0.825*	***0.989***	***0.981***	*−0.180*	*0.352*	*0.288*	*0.438*	*−0.774*	*0.587*	*−0.777*	*0.595*	*0.230*	*0.247*	*0.051*	*0.564*	*0.311*	*−0.717*	*−0.674*	*0.166*
Chl b	*−*0.132	*−***0.946**	0.568	**1**	***0.880***	***0.920***	*−0.304*	*0.552*	*0.659*	*0.013*	*−0.307*	*0.311*	*−0.652*	*0.510*	*0.219*	*0.749*	*0.236*	*0.323*	*0.566*	*−0.643*	*−0.811*	*−0.287*
T Car	0.492	*−*0.283	**0.925**	0.396	**1**	***0.992***	*−0.133*	*0.459*	*0.418*	*0.424*	*−0.697*	*0.543*	*−0.782*	*0.649*	*0.305*	*0.350*	*0.046*	*0.454*	*0.411*	*−0.702*	*−0.685*	*0.109*
T Chl	0.421	*−*0.699	**0.973**	0.742	0.864	**1**	*−0.231*	*0.436*	*0.429*	*0.309*	*−0.644*	*0.516*	*−0.766*	*0.590*	*0.236*	*0.432*	*0.118*	*0.504*	*0.412*	*−0.721*	*−0.750*	*0.015*
TSP	*−*0.673	0.628	*−*0.409	*−*0.357	*−*0.111	*−*0.433	**1**	*−0.055*	*−0.071*	*0.410*	*0.124*	*0.377*	*−0.286*	*0.008*	*0.131*	*−0.286*	*−****0.898***	*−0.492*	*0.477*	*0.661*	*0.766*	*0.095*
TPC	0.212	0.312	0.558	*−*0.102	0.776	0.426	0.438	**1**	***0.930***	*0.363*	*−0.121*	*−0.370*	*−0.002*	*0.877*	***0.894***	*0.463*	*0.387*	*−0.483*	*0.243*	*−0.565*	*−0.523*	*0.203*
Tan	0.387	*−*0.015	0.768	0.181	**0.952**	0.676	0.158	**0.899**	**1**	*0.050*	*0.143*	*−0.328*	*−0.082*	*0.672*	*0.683*	*0.741*	*0.340*	*−0.472*	*0.481*	*−0.416*	*−0.532*	*−0.170*
MDA	0.609	*−*0.487	0.815	0.487	0.849	0.800	*−*0.415	0.389	0.749	**1**	*−0.750*	*0.244*	*−0.241*	*0.727*	*0.668*	*−0.531*	*−0.242*	*−0.106*	*−0.115*	*−0.321*	*0.058*	*0.861*
SOD	*−*0.248	0.621	*−*0.653	*−*0.571	*−*0.353	*−*0.692	0.537	*−*0.067	*−*0.104	*−*0.179	**1**	*−0.459*	*0.443*	*−0.562*	*−0.289*	*0.395*	*−0.042*	*−0.550*	*0.243*	*0.663*	*0.379*	*−0.696*
TF	*−*0.060	0.780	*−*0.048	*−*0.615	0.157	*−*0.211	0.671	0.730	0.366	*−*0.312	0.127	**1**	*−****0.911***	*−0.095*	*−0.385*	*−0.082*	*−0.686*	*0.585*	*0.494*	*0.102*	*0.096*	*−0.100*
TOS	0.604	*−*0.665	**0.951**	0.650	0.863	**0.956**	*−*0.558	0.357	0.679	**0.921**	*−*0.547	*−*0.318	**1**	*−0.180*	*0.142*	*−0.263*	*0.517*	*−0.479*	*−0.704*	*0.126*	*0.196*	*0.199*
AA	*−*0.098	0.785	*−*0.121	*−*0.641	0.048	*−*0.278	0.645	0.640	0.244	*−*0.425	0.086	**0.990**	*−*0.399	**1**	***0.919***	*0.108*	*0.259*	*−0.226*	*0.076*	*−0.704*	*−0.499*	*0.563*
Sug	*−*0.737	0.647	*−*0.696	*−*0.497	*−*0.571	*−*0.706	0.799	0.031	*−*0.370	*−*0.872	0.330	0.609	*−*0.869	0.671	**1**	*0.021*	*0.257*	*−0.566*	*−0.026*	*−0.478*	*−0.256*	*0.586*
Amy	0.362	0.421	0.521	*−*0.269	0.713	0.350	0.343	**0.970**	0.832	0.325	*−*0.087	0.792	0.308	0.717	0.027	**1**	*0.283*	*−0.058*	*0.650*	*−0.210*	*−0.568*	*−0.745*
Prot	0.755	0.569	0.215	*−*0.638	0.377	*−*0.003	*−*0.052	0.518	0.486	0.326	0.251	0.477	0.167	0.421	*−*0.230	0.670	**1**	*0.097*	*−0.522*	*−0.700*	*−0.735*	*0.086*
Est	0.075	**0.918**	*−*0.203	*−*0.797	0.109	*−*0.388	0.661	0.639	0.382	*−*0.150	0.560	0.854	*−*0.354	0.809	0.456	0.706	0.706	**1**	*−0.141*	*−0.375*	*−0.415*	*−0.065*
Pro	*−*0.491	0.816	*−***0.905**	*−*0.768	*−*0.696	*−***0.951**	0.674	*−*0.160	*−*0.443	*−*0.730	0.787	0.412	*−***0.933**	0.448	0.794	*−*0.121	0.079	0.593	**1**	*0.238*	*−0.001*	*−0.601*
CAT	*−*0.094	0.280	0.441	*−*0.011	0.644	0.356	0.607	**0.945**	0.775	0.182	*−*0.113	0.762	0.204	0.692	0.260	0.872	0.244	0.562	*−*0.078	**1**	***0.906***	*−0.373*
POD	*−*0.054	0.694	0.085	*−*0.466	0.395	*−*0.061	0.757	0.874	0.638	0.008	0.324	**0.897**	*−*0.121	0.828	0.426	0.861	0.519	**0.896**	0.340	0.866	**1**	*0.054*
APX	*−*0.065	0.429	*−*0.596	*−*0.469	*−*0.368	*−*0.616	0.234	*−*0.270	*−*0.190	*−*0.037	**0.944**	*−*0.156	*−*0.406	*−*0.193	0.065	*−*0.270	0.254	0.348	0.633	*−*0.377	0.052	**1**

*Values in bold are different from 0 with a significance level alpha = 0.05; Values above diagonal in italic are under Drought condition and values below diagonal are under normanl conditions. **Legends:** Ly, Lycopene; β-Car, β-Carotene; Chl a, Chlorophyll a; Chl b, Chlorophyll b; T Car, total Carotenoids; T Chl, Total Chlorophyl; TSP, Total soluble protein; TPC, total phenolic contents; Tan, Tannins; MDA, Malondialdehyde; SOD, Sodiumoxide peroxidase; TF, Total Flavonoid; TOS, total oxidant status; AA, Ascorbic acid; Sug, Sugars; Amy, Amylase; Prot, Protease; Est, Esterase; Pro, Proline; CAT, Catalase; POD, peroxidase dismutase; and APX, ascorbate peroxidase.*

A positive and highly strong correlation was found for the following characters under drought conditions: chlorophyll a and lycopene (0.886); chlorophyll b and lycopene (0.988); total carotenoids and lycopene (0.935); total chlorophyll and lycopene (0.958); total carotenoids and chlorophyll a (0.989); total carotenoids and chlorophyll b (0.880); total chlorophyll and chlorophyll a (0.981); total chlorophyll and chlorophyll b (0.920); total chlorophyll and total carotenoids (0.992); MDA and beta-carotene (0.991); tannins and TPC (0.930); sugars and TPC (0.894); sugars and ascorbic acid (0.919); and CAT and POD (0.906). A negative and highly significant correlation was found in the following characters in drought conditions: TOS and TF (−0.911) and protease and TSP (−0.898; Table 8, values above diagonal in italic).

#### PCA for Biochemical Analysis

**FIGURE 9:**
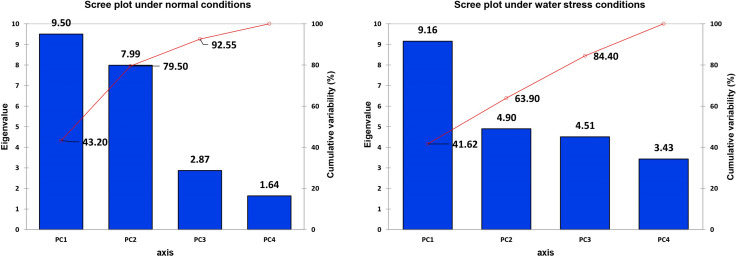
Scree plot between eigenvalues and factors under control and drought conditions for biochemical traits.

It is obvious from the scree plot (Figure 9) that four factors contributed for the total variation. All the four factors divulged eigen values ≥ 1 under normal and water-stressed conditions. Table 9 revealed that the PC 1 showed the maximum variability (43.20% and 41.62%) followed by PC 2 (36.30% and 22.28%), PC 3 (13.05% and 20.50%), and PC 4 (7.45% and 15.60%) under normal and water-stressed conditions, respectively.

**TABLE 9:** Principle component analysis of different biochemical traits in cotton under normal and drought conditions.

	Normal conditions				Drought conditions			
	PC1	PC2	PC3	PC4	PC1	PC2	PC3	PC4
Eigenvalue	9.50	7.99	2.87	1.64	9.16	4.90	4.51	3.43
Total variance (%)	43.20	36.30	13.05	7.45	41.62	22.28	20.50	15.60
Cumulative variance (%)	43.20	79.50	92.55	100.00	41.62	63.90	84.40	100.00

The characters like proline-N, TOS-N, total chlorophyll-N, total carotenoids-N, and sugars-N showed considerable positive contribution in PC 1, whereas PC 2 was associated with diversity among genotypes due to amylase-N, TPC-N, CAT-N, and POD-N with their positive contribution. The PC 3 explicated variation among cotton genotypes owing to protease-N, APX-N, and lycopene-N with positive influence. The PC 4 elucidated the variance among the genotypes for SOD-N, lycopene-N, and TSP-N with positive denominations (Table 10). The characters like total carotenoids-D, lycopene-D, and total chlorophyll-D showed substantial positive contribution in PC 1, whereas PC 2 was associated with diversity among genotypes due to APX-D, amylase-D, and MDA-D with their positive contribution. The PC 3 elucidated variation among cotton genotypes owing to TF-D, protease-D, and TOS-D with positive influence. The PC 4 explicated the variance among the genotypes for TSP-D, proline-D, and esterase-D with positive denominations (Table 11).

**TABLE 10:** Contribution of the variables (%) under control conditions.

	PC1	PC2	PC3	PC4
Lycopene-N	2.449	1.337	16.228	11.882
Beta-carotene-N	8.027	1.960	2.104	1.235
Chl a-N	7.478	3.540	0.131	0.168
Chl b-N	6.799	0.627	6.723	6.761
Total carotenoids-N	4.317	6.965	0.027	1.988
Total Chl-N	8.737	1.719	1.040	0.154
TSP-N	5.512	1.800	4.795	11.885
TPC-N	0.015	12.181	0.509	0.674
Tannins-N	1.548	9.553	0.156	5.207
MDA-N	6.381	1.757	4.958	6.765
SOD-N	4.871	0.020	8.315	18.111
TF-N	3.382	7.274	1.463	3.391
TOS-N	9.148	1.384	0.484	0.379
Ascorbic acid-N	3.908	5.729	2.176	6.624
Sugars-N	7.951	0.001	8.463	0.084
Amylase-N	0.002	12.381	0.000	0.662
Protease-N	0.145	5.205	17.318	4.482
Esterase-N	4.637	6.173	2.294	0.026
Proline-N	10.098	0.193	0.398	0.821
CAT-N	0.038	9.961	6.116	1.544
POD-N	2.097	9.603	0.195	1.712
APX-N	2.460	0.636	16.107	15.443

**TABLE 11:** Contribution of the variables (%) under drought conditions.

	PC1	PC2	PC3	PC4
Lycopene-D	9.292	2.820	0.029	0.281
Beta-carotene-D	3.135	12.419	2.065	0.326
Chl a-D	9.518	0.124	2.146	0.744
Chl b-D	8.584	4.127	0.175	0.111
Total carotenoids-D	10.124	0.249	1.324	0.030
Total chl-D	9.976	0.902	0.759	0.234
TSP-D	0.583	0.944	6.995	17.042
TPC-D	4.721	1.166	6.606	6.195
Tannins-D	3.771	0.272	7.581	8.724
MDA-D	2.152	13.000	2.995	0.897
SOD-D	4.694	4.474	4.062	4.887
TF-D	0.988	1.598	18.427	0.001
TOS-D	3.923	2.977	10.415	0.731
Ascorbic acid-D	6.882	5.272	0.962	1.985
Sugars-D	2.767	8.410	3.347	5.348
Amylase-D	1.761	11.326	4.467	2.397
Protease-D	0.659	0.053	14.983	7.612
Esterase-D	0.964	2.182	4.247	17.870
Proline-D	1.087	7.169	1.511	14.016
CAT-D	7.337	0.746	2.368	5.385
POD-D	6.703	1.025	4.208	4.258
APX-D	0.380	18.746	0.328	0.925

**FIGURE 10:**
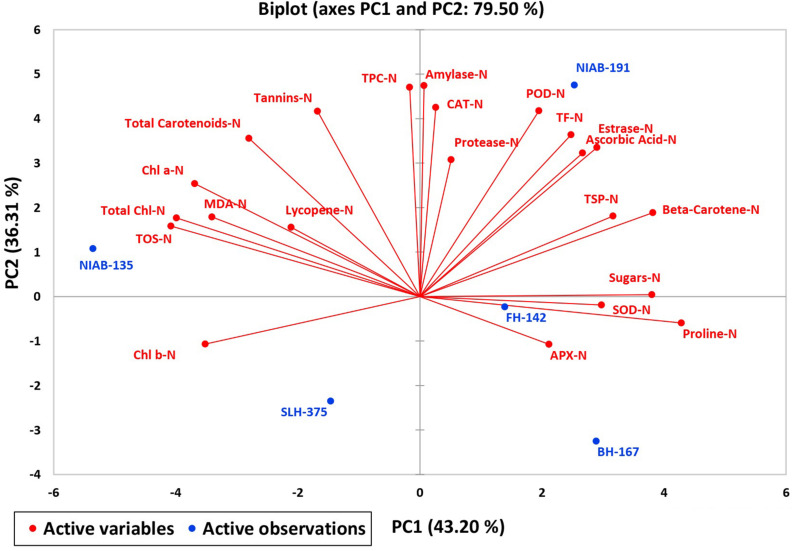
Biplot illustrating contribution of various traits under control conditions.

**FIGURE 11:**
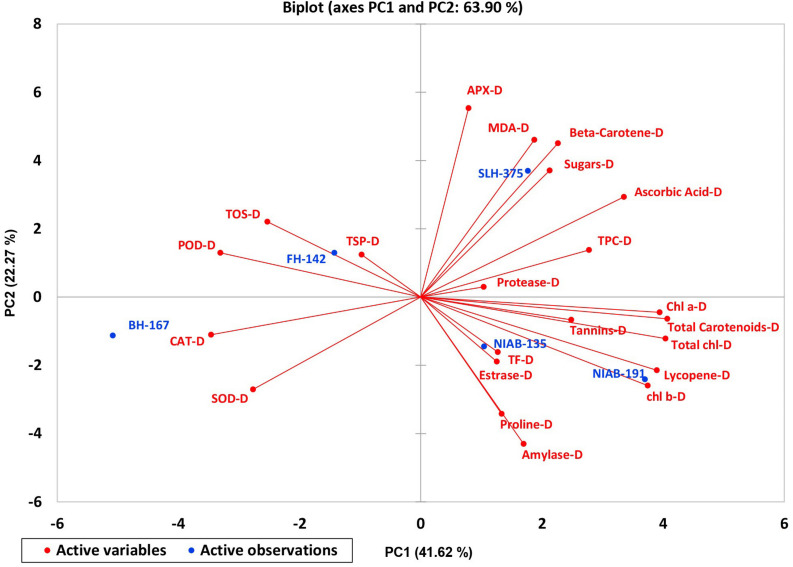
Biplot illustrating contribution of various traits under drought conditions.

A biplot between PC 1 and 2 (Figures 10, 11) divulged that traits and genotypes are superimposed on the plot as vectors under control and water-stressed conditions. It is evident from the distance of each trait with respect to PC1 and PC2 that these triats were responsible for the variation among the genotypes. The biplot revealed that SC-D, total carotenoids-N, β-carotene-N, proline-N, TOS-N, total carotenoids-D, chlorophyll a-D, CAT-D, lycopene-D, and chlorophyll b-D were contributors of variability in cotton germplasms under study. Moreover, PC 1 and PC 2 were responsible for 79.50 and 63.90% variation among the genotypes under normal and water-stressed conditions, respectively.

## Discussion

Drought tolerance in cotton genotypes requires two components: self-selection or natural selection. For tolerance induction, there must be variability in the characters of plants and characters that must be controlled by substantial additive components.

Twenty-three genotypes were collected and sown in the greenhouse   under   controlled   conditions  in  order  to  study  the behavior of plants and their variability at the seedling stage. We studied morphological and physiological characters, i.e., RL, SL, RWC, ELWL, transpiration rate, SC, and photosynthesis. We also analyzed the biochemical characters, i.e., chlorophyll a, chlorophyll b, total chlorophyll, β-carotene, total carotenoids, tannins, TPC, TOS, APX, POD, SOD, MDA, CAT, TF, and TSP. Panda et al.’s results showed that in tolerant genotypes, more compatible solutes were accumulated in comparison to susceptible genotypes. Reduction in RWC was detected in the leaf, which was recovered. It may be due to higher contents of sugars, polyphenols, proline, and amino acids, which are compatible solutes (Parida et al., 2007). Parida et al. (2007) found significant decrease in chlorophyll content, carotenoids, proteins, and starch after applying drought stress for 7 days. Generated data were compared using drought susceptibility indices, drought tolerant indices, and other absolute values.

A significant decrease in RLs was observed under drought conditions. Genotypes BH-167, FH-142, NIBGE-2, MNH-786, and CIM-663 were found to be susceptible under drought conditions on the basis of RL. The most tolerant genotypes to drought or moisture stress were NIAB-135, SLH-375, BH-160, CIM-554, and FH-498. In some studies, it has been supported that increase in RL enhances the plant potential to fetch water from deeper soil (Ludlow and Muchow, 1990), but at the same time, elongated roots may affect the shoot growth as more photosynthates move toward roots (Saleem et al., 2016). The percentage decrease observed in RL under drought conditions ranged from 7% to 51%, while few studies revealed that in some plants like tall fescue, roots were more sensitive toward drought conditions (Huang and Gao, 2000).

Under drought stress, a significant reduction was observed in shoot elongation. SLH-375, NIAB-135, and CIM-663 are drought tolerant on the basis of SL. NIAB-852, BH-167, and BH-160 failed to grow well under drought stress. The percentage decrease in SL ranged from 97.14 to 35.17%. It is reported that the decrease in shoot or RL is due to imbalance observed in water relations (Simonneau et al., 1993). The level of ABA concentration increases due to water stress. ABA is in fact shoot growth inhibitor (Achard et al., 2006), and this causes reduction in shoot growth. It is observed that shoots were more sensitive to drought stress in comparison to roots. This study results endorsed what previous studies have done (Govindaraj et al., 2010; Iqbal et al., 2011).

Morphological characters like ELWL and RWC played a substantial role in the differentiation of drought-tolerant varieties from the others. When we talk about ELWL, genotypes that show the lowest values are required, due to minimum water loss under drought. ELWL exhibits the cuticle thickness as, after detachment from the plant, water transpires through the epidermis (Saleem et al., 2016). ELWL and transpiration are vital selection traits for tolerance to water shortage as they are controlled by the cuticle layer thickness and waxiness (Rahman et al., 2000). On the basis of ELWL, NIAB-512, MNH-786, CIM-663, and CIM-554 are the genotypes that showed minimum water loss in stressed conditions, and the susceptible genotypes that showed maximum water loss were NIAB-852, NIAB-846, and BH-167. The percentage decrease in ELWL ranged from 14 to 81%.

Relative water content is an indicator of water status in plant leaf and it is also a good trait for the identification of drought stress (Sánchez-Blanco et al., 2002). RWC is affected by physiological characters (Kramer and Boyer, 1995). RWC also decreases under the influence of drought conditions (Ullah et al., 2012). A similar pattern of RWC was reported in wheat crops (Hasheminasab et al., 2012). High WC has been considered as an efficient screening parameter for drought-tolerant genotypes in barley (Matin et al., 1989), *Triticum aestivum* (Geravandi et al., 2011), and fescue (Huang and Fry, 1998). Notable drought tolerance in NIAB-512, NIAB-135, VH-363, and FH-498 was observed as they maintained a high proportion of RWC. On the other hand, BH-167, MARVI, and BH-160 seemed to be unfortunate retainers of RWC. The percent reduction of RWC was 6–22%.

Photosynthesis and SC decrease under drought conditions due to the closure of stomata and the defense mechanism adopted by plants in order to prevent water loss. Reduced photosynthesis is due to a decrease in CO_2_ diffusion to carboxylation sites in plants (Lawlor and Cornic, 2002). At the same time, in high temperatures and less water supply, the SC also reduces (Carmo-Silva et al., 2012). Despite being helpful in preventing water loss, it also affects the CO_2_ influx, ultimately decreasing photosynthesis (Flexas et al., 2004); 2–61% decrease in SC was observed in our experiment, whereas in the case of photosynthesis, the decline observed was 0.74 to 19.8%.

Drought susceptibility indices give an inside view of the whole crop response to water stress. That is why drought indices play a vital role in the selection of genotypes with high potential of yield, for example, *Abelmoschus esculentus* (Naveed et al., 2010), common bean (Porch, 2006), and *Triticum aestivum* L. (Golabadi et al., 2006). NIAB-135, NIAB-512, and CIM-554 proved to be tolerant to water stress. They have a high genetic potential for excellent working under drought stress conditions.

Under a short supply of water, the leaf functions are highly affected and the harmful products start producing at enormous speed, i.e., ROS. This is due to the imbalance between light capture and its utilization by the plant systems, as a result of which, superoxide anion, hydroxyl radicals, singlet oxygen, and H_2_O_2_ production occurs (Munneâ-Bosch and Penäuelas, 2003). ROS attacks the cell machinery, as a result of which, the activities of the cell system are disturbed (Reddy et al., 2004). Nevertheless, plants have evolved and developed their machinery to cope with ROS. The production of ROS in cotton under drought has been reported in previous studies. At the same time, APX production also increased that helped the plants to scavenge the ROS (Ratnayaka et al., 2003). APX was also increased in our genotypes. The most tolerant genotypes, i.e., NIAB-135 and SLH-375 showed more increase in APX content under drought stress.

Superdioxide dismutase content in drought-susceptible genotypes, i.e., BH-167 and FH-142, was found to increase under drought stress. SOD activity has been reported to increase under drought stress at the seedling stage (Ahmadi et al., 2010). MDA content was found to decrease under drought stress except for SLH-375, a tolerant genotype that showed a significant increase in MDA content. It reflects that other varieties failed to cope with the rapid production of ROS. The same trend of susceptibility has been reported in previous studies (Hafeez et al., 2015).

Proline and total soluble proteins were found to increase in all varieties under drought stress. It has been reported that proline and TSP either increase or decrease under environmental stresses (Parida et al., 2004). Proline not only acts as an osmolyte but also contributes to stabilizing subcellular level structures (e.g., membranes and proteins), scavenging free radicals, and buffering cellular redox potential under stress conditions (Iqbal, 2009). Higher proline content was reported in drought-tolerant species of cotton, tall fescue, and wheat (Man et al., 2011; Sultan et al., 2012). In this study, we observed an increase in proline content in our all varieties except for FH-142, in which a slight decrease in proline content under drought stress was observed. In the case of TSP content, all varieties exhibited a decrease under drought stress. Only SLH-375 showed an increase in TSP content. Overall, the activity of enzymes (POD, CAT, APX, and SOD) involved in ROS scavenging increased significantly under drought conditions (Hasan et al., 2018). The response of different varieties toward different enzymatic and non-enzymatic activities may vary.

## Conclusion

At present, drought is the major constraint to crop yield and is posing threat to the future of agriculture, so it is necessary to develop drought-tolerant as well as high-yielding varieties. With the help of performing morphological, physiological, and biochemical analysis, i.e., RL, SL, SC, photosynthesis, proline, CAT, MDA, and lycopene, we were able to identify the varieties that are better suited to water shortages and could germinate and grow better in harsh environments. These varieties could cope with drought effects by adapting to the changes that occurred due to water losses. Identification of such traits that could distinguish tolerant versus susceptible varieties is encouraging as they are easy to analyze and could help us in screening a gene pool for genes of our interest. A wide range of diversity was found in all the physiological, biochemical, and morphological traits, implying that we can do the selection of varieties as drought-tolerant and susceptible ones. By concluding all the results, we found that out of our 23 experimental varieties, NIAB-135, NIAB-512, and CIM-554 could be used for breeding strategies for the development of drought-tolerant varieties owing to their drought tolerance as well as the high genetic potential for better performance under drought stress.

## Data Availability Statement

The original contributions presented in the study are included in the article/supplementary material, further inquiries can be directed to the corresponding author/s.

## Author Contributions

MK, AH, and ZZ designed the experiments. ZZ and AD carried out the majority of the experiments. ZZ, MK, AH, and AD carried out all computational analyses. MA, ZZ, and GF carried out the physiological aspect of the experiment. ZZ and AH performed the biochemical analysis. ZZ and HH participated in glasshouse experiments. MK and AH supervised the experiments. ZZ and AD drafted the manuscript. MK, AH, and MA revised the manuscript. All authors read and approved the final manuscript.

## Conflict of Interest

The authors declare that the research was conducted in the absence of any commercial or financial relationships that could be construed as a potential conflict of interest.

## Footnotes

1 http://www.xlstat.com
